# Administration of oxytocin antagonist at the same time as using a Foley catheter with cotton swab before embryo transfer for cervical stenosis

**DOI:** 10.1186/s12905-021-01409-y

**Published:** 2021-07-03

**Authors:** Heesuk Chae

**Affiliations:** Department of Obstetrics and Gynecology, Research Institute of Clinical Medicine of Jeonbuk National University, Biomedical Research Institute of Jeonbuk National University Hospital, Jeonbuk National University, 20, Geonji-ro, Deokjin-gu, Jeonju-si, Jeollabuk-do 561-712 South Korea

**Keywords:** Embryo transfer, Cervical stenosis, Foley catheter, Atosiban

## Abstract

**Background:**

Embryo transfer without difficulty in a patient with cervical stenosis can be a great challenge for in vitro fertilization (IVF). We report a successful pregnancy following a frozen thawed embryo transfer after administration of an oxytocin antagonist at the same time as using a Foley catheter with cotton swab in a patient with refractory cervical stenosis.

**Case presentation:**

A 40-year-old woman undergoing IVF. The patient’s previous embryo transfers were difficult. For every transfer, uterine manipulation was needed, force was required, and dilatation was necessary. A Foley catheter with a cotton swab was inserted into the cervical canal, atosiban was administered at the same time, and the Foley catheter was removed immediately before embryo transfer. A smooth transfer was performed without bleeding, force, uterine manipulation, or cervical dilator. The patient became pregnant and delivered by cesarean section at term.

**Conclusion:**

This method is effective in performing atraumatic embryo transfer in patients with cervical stenosis.

## Background

The embryo transfer (ET) without difficulty has been recognized as one of the most critical factors in the success of in vitro fertilization (IVF) cycles. A difficult transfer usually result in bleeding with multiple attempts and, in more difficult cases, even cervical dilators can be used. Blood on the outside of the catheter at the time of ET and multiple attempts of catheter entry have been associated with decreased pregnancy rates [1]. ET using additional maneuvers such as tenaculum has been shown to progressively decrease pregnancy rate [2]. In the end, atraumatic transfer technique in a patient with cervical stenosis can be a great challenge for IVF.

The present case report describes a successful pregnancy following a frozen thawed embryo transfer after administration of oxytocin antagonist at the same time as using a Foley catheter with cotton swab in a patient with refractory cervical stenosis.

## Case presentation

The patient is a 40-year-old, gravida 0, para 0, who presented with two years of infertility. The patient was diagnosed with type 2 diabetes mellitus at the age of 20 and is currently taking medication. She had a history of laparoscopic myomectomy a year ago. Menstrual cycles had been regular with normal flow. Transvaginal ultrasonography showed that the anteverted and anteflexed uterus and both ovaries were normal. Baseline hormonal assays showed the following: follicle stimulating hormone (FSH) 7.2 mIU/mL, luteinizing hormone (LH) 11.5 mIU/mL, estradiol (E_2_) 30.4 pg/mL, progesterone (P) 0.35 ng/mL. Serum anti-Müllerian hormone (AMH) level was 4.1 ng/mL. The hysterosalpingography (HSG) and semen analysis were unremarkable. She had received several cycles of timed intercourse at local clinic before coming here and two cycles of ovulation induction with clomiphene citrate at our clinic, but all failed. After counseling, she was scheduled for a long protocol of down-regulation using gonadotropin-releasing hormone (GnRH) agonist. For oocyte maturation, human chorionic gonadotropin (hCG) was given when the leading follicle was more than 18 mm. Nineteen oocytes were retrieved by an ultrasonic-guided transvaginal route 36 h later, of which 11 fertilized. After filling the bladder, a clear line from the cervix to the endometrium was confirmed by ultrasound. Embryo transfer was attempted using a Wallace catheter with stylet (Smiths Medical, Norwell, MA, USA) under transabdominal ultrasound guidance 5 days after oocyte pickup. However, the entrance into the external cervical os was not possible. Therefore, after holding the cervix with an Allis forcep, the catheter was tried again, but failed. We tried again with a sound and it was very difficult to successfully enter. After removing the sound, we tried to enter the catheter again, but we failed, so we used a Hegar dilator of 3 mm diameter (Hegar number 1) to widen the entrance of the cervix. After removing the Hegar dilator, the Wallace catheter passed through the internal cervical os, but stopped at about 1 cm from the internal os and did not proceed further. We widened a Hegar dilator of 4 mm diameter (Hegar number 2) to insert the catheter using a sound as a guide. However, the sound went close to the uterine fundus, but the catheter was no longer accessible close to the internal os. Eventually, the Hegar number 1 lifted the catheter and changed direction, allowing it to enter the uterus. The procedure took almost an hour. Three embryos were transferred, but pregnancy was unsuccessful.

**Fig. 1:**
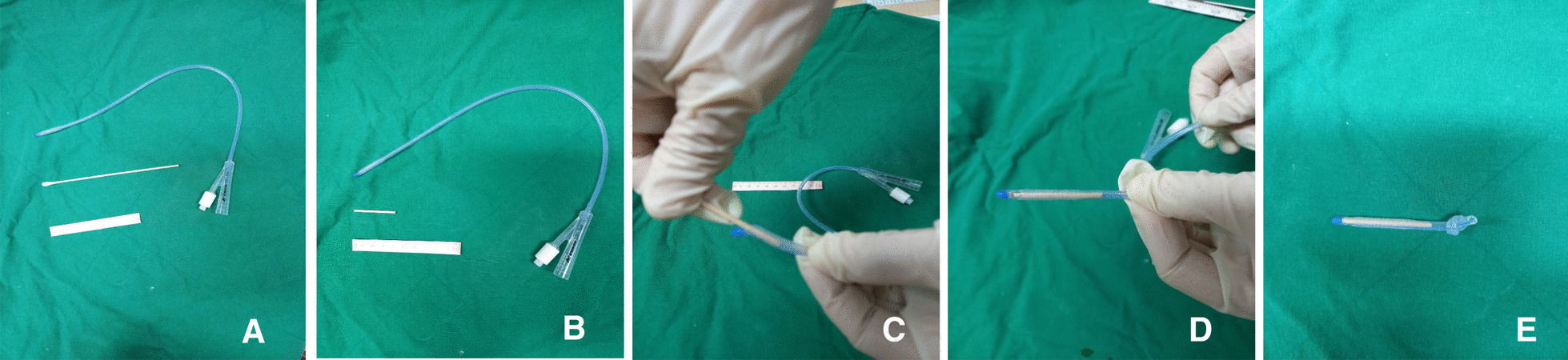
The steps to make a Foley catheter with cotton swab are shown in order from left to right

**Fig. 2:**
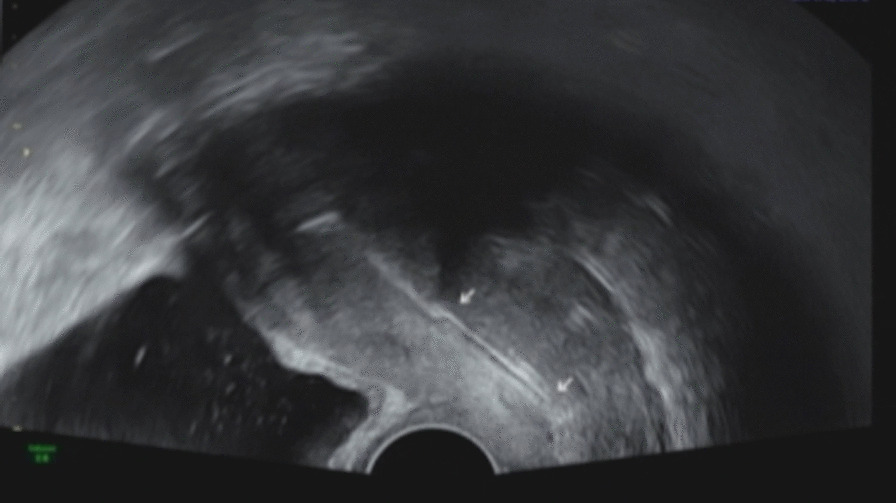
Transvaginal US scan showing the Foley catheter with cotton swab inserted into cervical canal

**Fig. 3:**
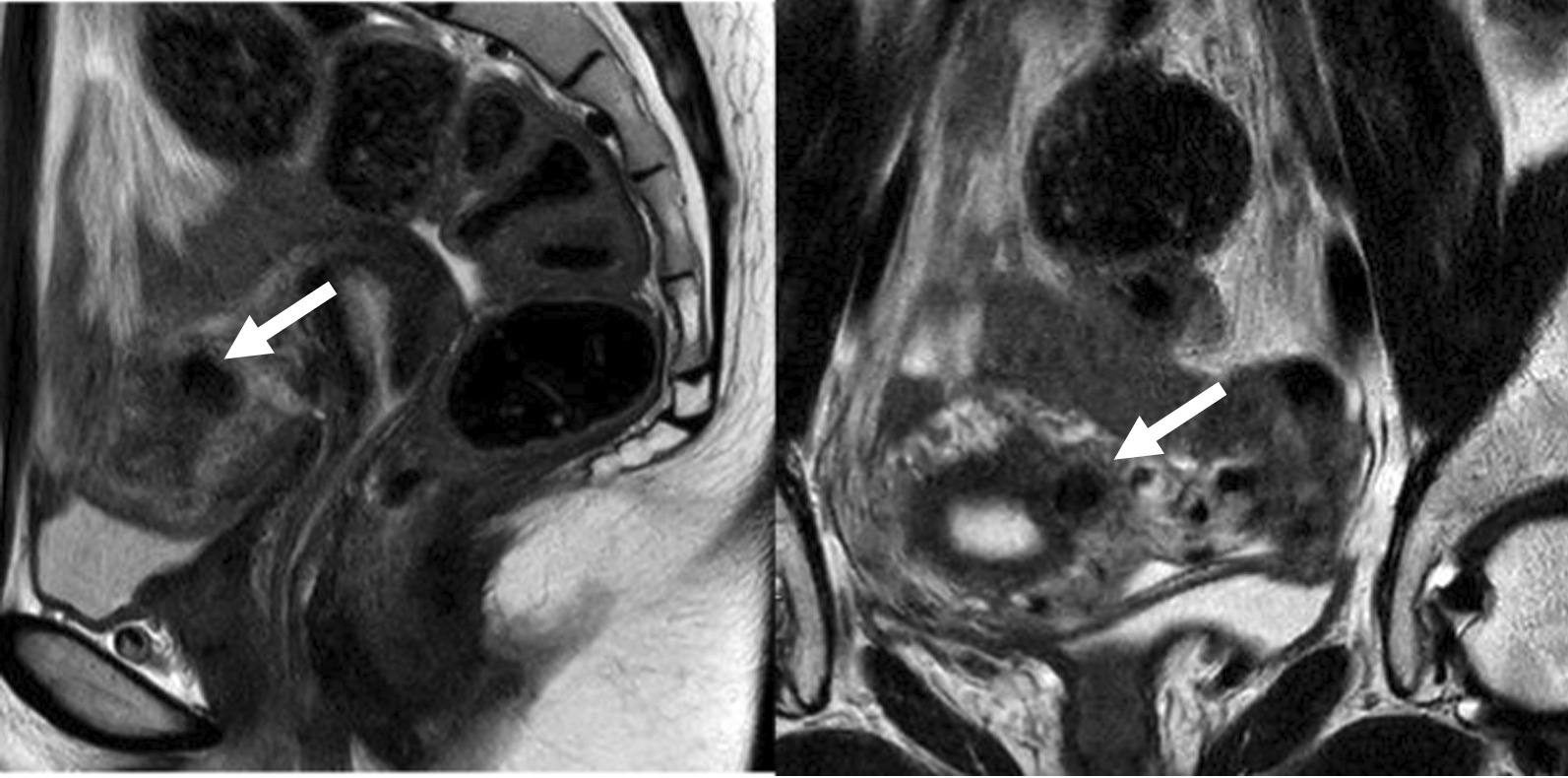
MRI showing an intramural fibroid in contact with the endometrium

Frozen embryo transfer (FET) started with administration of GnRH agonist (decapeptyl 0.1 mg; Ferring, Saint-Prex, Switzerland) in the mid luteal phase of the preceding cycle followed by administration of oral estradiol valerate (progynova 6 mg/day; Bayer Schering Pharma, Berlin, Germany) daily starting on cycle-day 3. Vaginal sonography showed endometrial thickness of 8.3 mm with trilaminar pattern. Therefore, GnRH agonist was stopped and 100 mg vaginal progesterone (Lutinus; Ferring Pharmaceuticals) two times daily was administrated until the day before embryo transfer. To facilitate embryo transfer, it was planned to use of a Foley catheter with the cotton swab after oral administration of misoprostol (Cytotec; Ali Raif, Istanbul, Turkey). After taking 200 µg of misoprostol orally twice every six hours, the procedure was performed 6 h after taking the last misoprostol. After inserting the wooden part of the cotton swab through the bladder opening of a 13-Fr Foley catheter, when it reached the length of the cervical canal, a knot was made and the rest was cut off (Fig. 1). The Foley catheter easily passed through the cervix and stopped due to the knot. Transvaginal ultrasound was confirmed that the Foley catheter was well inserted from the cervix to the internal os (Fig. 2). After removing the Foley catheter after 24 h, it was confirmed that a Hegar number 1 passed well. However, the entrance of the Wallce catheter into the uterus was not possible at the time of embryo transfer after 2 days. The same situation was repeated as in the first cycle. The embryos were difficultly transferred, but the pregnancy was unsuccessful. Magnetic resonance imaging (MRI) was performed to determine the cause of the catheter not proceeding anymore after entering the catheter 1 cm above the internal os. On MRI, a fibroid of about 1 cm in size was identified at the location (Fig. 3). After discussion, it was decided to continue the FET. The next FET was performed according to the protocol of the previous FET, but only Foley catheterization was performed 2 days before embryo transfer, and at the same time, atosiban (Tractocile; Ferring Arzneimittel, Kiel, Germany) was administered with an initial bolus dose of 6.75 mg followed by a high dose rate of 24 ml/hour during 3 h then reduced to 8 ml/hour up to 45 h. It had been planned that the Foley catheter with cotton swab would stay in situ and be removed immediately before embryo transfer. After removing the catheter, embryo transfer was easily performed using Cook^®^ Sydney IVF (Cook Medical, USA). The serum hCG on day 13 after embryo transfer was at 927.2 IU/L. The patient had no specific findings on antenatal care except for polyhydramnios. She delivered a healthy female baby weighing 3870 g by cesarean-section delivery.

## Discussion and conclusions

The conventional ET procedure is usually performed by transferring embryos through the cervix. Whether a transfer catheter is smoothly introduced through the cervical canal without force or trauma up to 1–2 cm from the fundus is known to be an important limiting factor in pregnancy success following IVF [3–5]. Several factors related with the positive correlation of difficult ET and reduced pregnancy rates have been suggested. The presence of blood on the transfer catheter decreased the likelihood of implantation and eventually reduced pregnancy rate [6]. Goudas et al. reported that blood found outside, but not inside, the embryo transfer catheter was associated with lower embryo implantation and clinical pregnancy rates [1]. Another factor that difficult ET can decrease pregnancy rates is the stimulation of uterine contractions. Manipulating with a tenaculum to the cervix or touching the uterine fundus induces uterine contractions by secreting prostaglandins (PG) and oxytocin [7, 8]. Fanchin et al. showed that uterine contractions at the time of ET was associated with a decrease in implantation rates and clinical pregnancy rates [9]. Wood et al. demonstrated that performance of ET with a soft catheter under ultrasound guidance increased in clinical pregnancy rates [10].

Several attempts have been made to overcome such situations as cervical stenosis, where difficult transfers are expected. These include trying with a mock or dummy embryo catheter before ET [4, 11], straightening the cervicouterine angle by pulling the cervix with a tenaculum or passive filling the bladder [12, 13], or using ultrasonographic guidance during the ET [14]. In addition, when cervical dilatation method was performed during an ovum pick–up [15], at the initial visit in an IVF-ET cycle (approximately 2 weeks before ET) [16], 1–3 months before ET [17], or using laminaria tents [18], ET became easier and the pregnancy rate improved. Hysteroscopic revision of the cervical canal resulted in easier ET and improved pregnancy rates in patients with cervical stenosis [19]. However, although the above methods may be useful for the ET, the procedure is still difficult or impossible for some patients. Furthermore, while reporting the occurrence of uterine perforation or cervical incompetence in the cervical dilatation method, the authors reported that attention should be paid to the risks [17]. It was noted that hysteroscopic revision of the cervical canal also needed to be counseled regarding potential obstetric risks [20]. Ultrasound-guided, transmyometrial ET has been suggested as an alternative method to transcervical ET [21, 22]. However, according to the study comparing ultrasound–guided transmyometrial and transcervical ET, there was no benefit from transmyometrial ET compared to conventional transcervical ET in patients with cervical stenosis or in patients who had failed to conceive in previous cycles [23]. Another study reported that transmyometrial ET is a good alternative option in cases of very difficult transcervical ET, but its superiority over conventional transcervical ET cannot be demonstrated [24]. The limitation of this method is that it is easily affected by the location of the uterus and the accuracy of ultrasound because the success of the procedure depends on how accurately the needle through uterine wall is approached to the uterine cavity [22].

In the present case, we used a Foley catheter with cotton swab to secure enough space for the implantation catheter to fit. We did not use laminaria tents because of concerns about the possibility of intrauterine infection [18] and the possibility of uterine contraction due to excessive dilatation of the cervix. Norman et al. have shown that misoprostol increases in the amplitude and frequency of uterine contractions [25]. Also, Fanchin et al. have reported that uterine contractions can be triggered by cervical manipulation [9]. As our case, cervical manipulation such as keeping a Foley catheter in the cervical canal may also cause uterine contractions. Moraloglu et al. have suggested that atosiban treatment before ET is effective in priming of the uterus for implantation, showing that atosiban increased the implantation rate and the clinical pregnancy rate [26]. As shown in our case, it is important that the dilatation of cervix is maintained until immediately before ET, and it may be an effective method for patients with cervical stenosis to give uterine relaxant together to suppress uterine contraction.

In conclusion, we present a case of successful pregnancy in a patient with cervical stenosis using a Foley catheter with cotton swab and concurrent oxytocin antagonist before ET. In case of cervical stenosis, it is important to keep the dilatation of cervix until immediately before ET, and it is recommended to use uterine relaxant together to prevent uterine contraction. In addition, the risk of obstetric complications such as cervical incompetence may be very low by using a thin Foley catheter. Further studies are needed to prove its effectiveness.

## Data Availability

Data and material are available on request from the corresponding author.

## Abbreviations

ET: Embryo transfer
IVF: In vitro fertilization
FSH: Follicle stimulating hormone
LH: Luteinizing hormone
E_2_: Estradiol
P: Progesterone
AMH: Anti-Müllerian hormone
HSG: Hysterosalpingography
GnRH: Gonadotropin-releasing hormone
hCG: Human chorionic gonadotropin
FET: Frozen embryo transfer
MRI: Magnetic resonance imaging
PG: Prostaglandins
IVF-ET: In vitro fertilization-embryo transfer
